# Isolated Vitamin D Deficiency Is Not Associated with Nonthyroidal Illness Syndrome, but with Thyroid Autoimmunity

**DOI:** 10.1155/2015/239815

**Published:** 2015-01-12

**Authors:** Muyesser Sayki Arslan, Oya Topaloglu, Bekir Ucan, Melia Karakose, Basak Karbek, Esra Tutal, Mustafa Caliskan, Zeynep Ginis, Erman Cakal, Mustafa Sahin, Mustafa Ozbek, Tuncay Delibasi

**Affiliations:** ^1^Department of Endocrinology and Metabolism, Diskapi Training and Research Hospital, 06330 Ankara, Turkey; ^2^Department of Biochemistry, Diskapi Training and Research Hospital, 06330 Ankara, Turkey; ^3^Department of Endocrinology and Metabolism, School of Medicine, Ankara University, 06230 Ankara, Turkey; ^4^Translational Research Center, Diskapi Teaching and Research Hospital, 06330 Ankara, Turkey; ^5^Department of Internal Medicine, School of Medicine (Kastamonu), Hacettepe University, 06330 Ankara, Turkey

## Abstract

*Aim*. This study aimed to compare thyroid functions, thyroid autoantibodies, and the existence of nonthyroidal illness syndrome (NTIS) according to vitamin D level. *Materials and Methods*. The study included age- and BMI-matched healthy volunteers with and without vitamin D deficiency. In addition, the nonthyroidal illness syndrome status was evaluated. *Results*. Anti-TPO positivity was significantly more common in those with severe and moderate vitamin D deficiency, as compared to those with a normal 25(OH)D level. Furthermore, TSH levels were significantly lower in those with severe and moderate vitamin D deficiency than in those with a normal 25(OH)D level. In addition, there was a significant weak inverse correlation between anti-TPO positivity and the 25(OH)D level and a positive correlation between the TSH level and 25(OH)D level. Only 1 thyroid function test result was compatible with NTIS among the participants with moderate vitamin D deficiency; therefore the difference was not significant. *Conclusions*. The prevalence of thyroid autoantibody positivity was higher in those with severe and moderate vitamin D deficiency than in those with a normal 25(OH)D level. Additional large-scale studies must be conducted to determine if vitamin D deficiency plays a causal role in the pathogenesis of Hashimoto's thyroiditis and NTIS.

## 1. Introduction

The best-known roles of vitamin D are in calcium metabolism and bone health; however, accumulating evidence shows that vitamin D has a variety of pro- and anti-inflammatory effects on the development of cancers, autoimmune diseases, and cardiovascular disorders. The biologically active form of vitamin D, 1,25-dihydroxyvitamin D [1,25(OH)_2_D3], controls genes responsible for cellular differentiation, proliferation, apoptosis, and angiogenesis, inhibits the production of T helper 1 cell cytokines, interleukin-2, interferon-*γ*, interleukin-5, tumor necrosis factor-*α* (TNF-*α*), and dendritic cell-dependent T-cell activation, and induces B-cell proliferation [1–3]. In T helper 2 cells vitamin D increases both the proliferation of interleukin-4 and transforming growth factor that suppresses inflammatory T-cell activation [1, 2, 4]. These interactions are important in the pathogenesis of Hashimoto's disease. Such proinflammatory cytokines as interleukin-6, TNF-*α*, and interleukin-1 have been suggested to be mediators in the pathogenesis of nonthyroidal illness syndrome (NTIS) [5]. In healthy volunteers, infusion of TNF-*α* caused thyroid-stimulating hormone (TSH) and triiodothyronine (FT3) levels to decrease [6]. A study on the effect of vitamin D on monocyte expression of TNF-*α* showed that 1,25(OH)_2_D3 could significantly suppress TNF-*α*, which plays an important role in the pathogenesis of autoimmune diseases [7]. Kankova et al. reported that serum peak levels of TNF-*α* were observed in mice fed a vitamin D-deficient diet [8]. The selenoenzyme family of iodothyronine deiodinases regulates the activity of thyroid hormones in many tissues and plays an important role in the pathogenesis of NTIS [5].

The presence of type-2 5′-deiodinase (D2) activity throughout the mouse skeleton in MC3T3-E1 cells and its induction by 1,25(OH)_2_D3 indicate that there is a relationship between thyroid hormone and 1,25(OH)_2_D3, demonstrating that D2 controls the differentiation or function of bone cells [9]. The majority of circulating T3 in the euthyroid state originates from muscle D2 and its impairment promotes a rapid decrease in T3 in those with NTIS [10]. In addition, in dying ICU patients D2 activity was negative in skeletal muscle [11]. The mortality rate was reported to be higher in those with vitamin D deficiency—in both the general population and ICU patients—and in critical care patients with NTIS [12–14]; however, no study has investigated the association between vitamin D deficiency and NTIS in ICU patients.

Considering that Hashimoto's disease is among the most commonly diagnosed autoimmune endocrine diseases and that thyroid function and the frequency of NITS in those with isolated deficiency of vitamin D have not been adequately studied, the present case-control study aimed to compare thyroid function test results, thyroid autoantibodies, and the existence of NITS according to vitamin D level.

## 2. Materials and Methods

The present study included healthy volunteers that presented to the hospital for routine check-up. Participants with a history of any metabolic, chronic, or systemic disease and those that were taking any medication that could affect thyroid function test results and/or the 25(OH)D level were excluded from the study. All the participants underwent physical examination, anthropometric measurement, and biochemical screening. The study population included 155 volunteers divided into 3 groups: group 1 included 53 volunteers (49 female, 4 male) with a vitamin D level < 10 ng mL^−1^; group 2 included 61 volunteers (51 female, 10 male) with a vitamin D level of 10–19.9 ng mL^−1^; group 3 included 41 volunteers (31 female, 10 male) with a vitamin D level ≥ 20 ng mL^−1^ [15]. The study was performed between December 2011 and February 2012. The Institutional Board Review evaluated and approved the study.

Serum samples were collected following overnight fasting for routine renal, hepatic, and thyroid function test, lipid profile, insulin level, and thyroid autoantibodies analysis. The fasting serum insulin level was evaluated using the chemiluminescent immunoassay method (Advia Centaur XP, Siemens Healthcare Diagnostics Inc., Tarrytown, USA). Insulin resistance was estimated via HOMA-IR calculation. Thyroid function tests were assessed for free FT3, free thyroxin (FT4), and TSH via chemiluminescent microparticle immunoassay (Abbott, Architect i2000, Abbott Laboratories Diagnostics Division, IL, USA). Antithyroglobulin antibody (anti-Tg) and antithyroid peroxidase (anti-TPO) levels were measured via chemiluminescent competitive immunoassay (Siemens, Advia Centaur XP). Normal ranges were as follows: FT3: 1.71–3.71 pg L^−1^; FT4: 0.7–1.48 ng dL^−1^; and TSH: 0.35–4.94 *μ*IU mL^−1^. Anti-Tg concentration > 6 IU mL^−1^ and anti-TPO concentration > 57 IU mL^−1^ were considered positive. The serum 25(OH)D3 level was measured using a commercial ELISA kit (Immuno-Biological Laboratories, Minneapolis, USA) with a normal range of 11.1–42.9 ng mL^−1^. A vitamin D level < 10 ng mL^−1^ was considered severe vitamin D deficiency (group 1), 10–19.9 ng mL^−1^ was considered moderate vitamin D deficiency (group 2), and ≥20 ng mL^−1^ was considered a normal vitamin D level (group 3). 

*Statistical Analysis.* Descriptive statistics were computed as mean ± SD (standard deviation), count, and percent frequencies. Kolmogorov-Smirnov test was used for normality test of numerical variables. One-Way ANOVA was used to determine group differences in numerical variables which have normal distribution, and significant group differences were determined via Tukey's post hoc test. In addition Kruskal-Wallis test was used for differences among the groups with regard to nonnormal numerical variables followed by nonparametric Tukey post hoc test. The relations between categorical variables and groups were investigated using the suitable Chi-square test. The correlation between continuous variables was evaluated by Spearman's or Pearson's correlation analysis where applicable. The level of statistical significance was set at *P* = 0.05 and PASW SPSS v.18.0 for Windows was used for all statistical computations.

## 3. Results

The clinical characteristics of the study participants according to group are shown in Tables 1 and 2. The number of males was higher in group 3 and this was considered during statistical analyzing. The groups were homogeneous in terms of mean age, BMI, HOMA-IR score, and smoking status, and there was not a significant difference in the lipid profile between the groups. The TSH levels differed significantly between groups (group 1: 1.2 ± 0.15 mIU mL^−1^; group 2: 1.5 ± 0.1 mIU mL^−1^; group 3: 1.9 ± 0.1 mIU mL^−1^) (*P* = 0.008); however, there were not any significant differences in the FT4 or FT3 levels between groups (*P* > 0.05). We also determined the NITS status of the participants. NTIS was noted in only 1 participant in group 2, which was not significant (*P* > 0.05). Anti-TPO positivity occurred most frequently in group 2 (71.7%), followed by group 1 (62.7%) and group 3 (24.2%) (*P* = 0.000); however, the frequency of anti-Tg positivity was similar in all 3 groups (*P* > 0.05).

**Figure 1:**
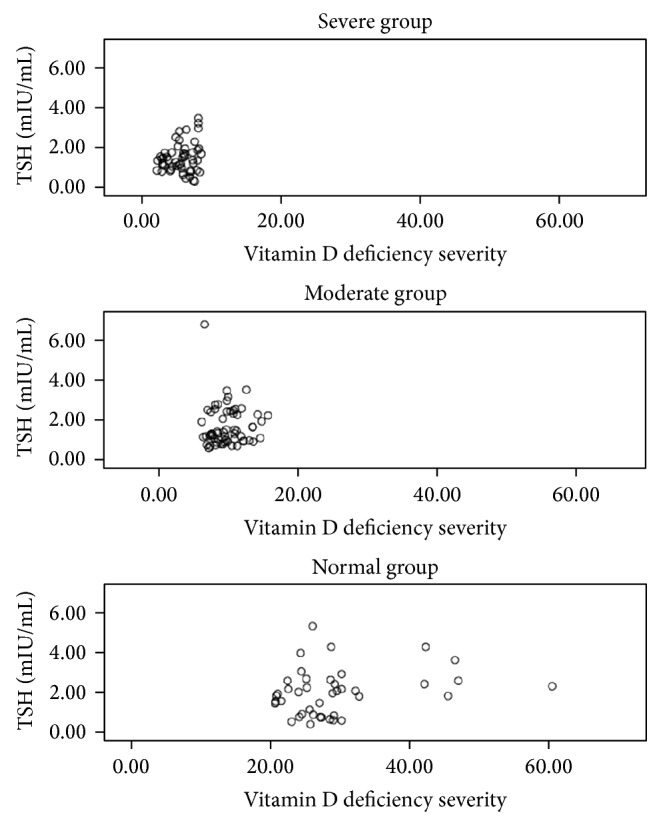
The correlation between TSH and vitamin D in each group.

**Figure 2:**
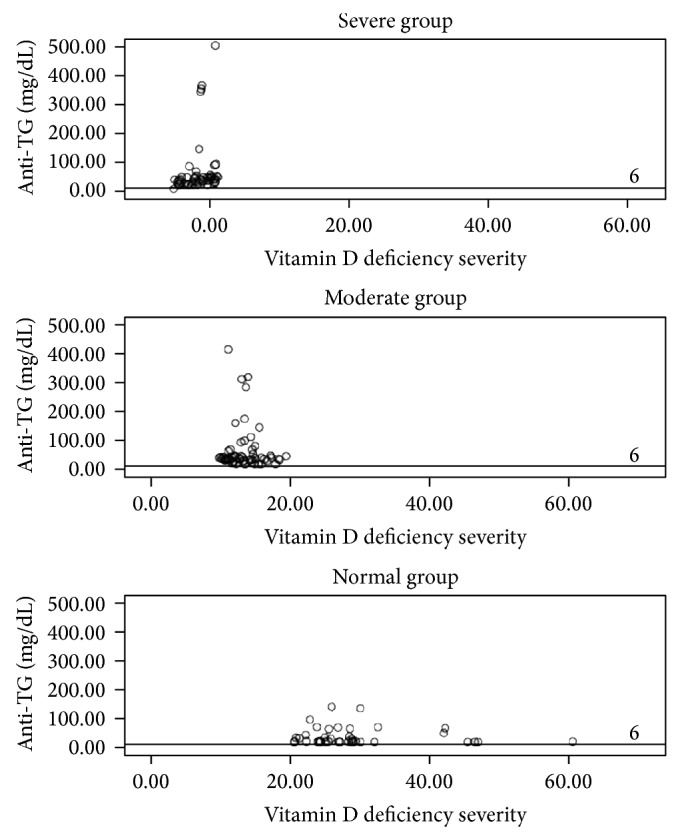
The correlation between anti-TG and vitamin D in each group. The cut-off value for negative anti-Tg concentration is <6 IU mL^−1^.

**Figure 3:**
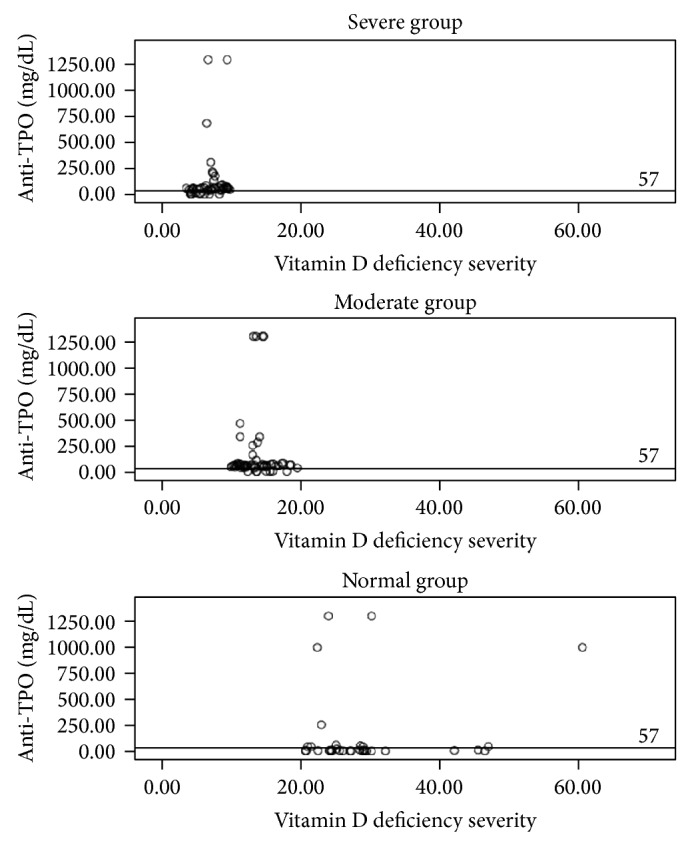
The correlation between anti-TPO and vitamin D in each group. The cut-off value for negative anti-TPO concentration is <57 IU mL^−1^.

A positive correlation was observed between the 25(OH)D3 and TSH levels; the TSH level increased significantly as the 25(OH)D3 level increased (*r* = 0.213, *P* = 0.008) (Figure 1). Furthermore, a negative correlation was observed between anti-TPO and anti-Tg levels and the 25(OH)D3 level (*r* = −0.199 and *P* = 0.017 and *r* = −0.154 and *P* = 0.05, resp.) (Figures 2 and 3).

## 4. Discussion

The most important finding of the present study is that the prevalence of thyroid autoantibody positivity in healthy volunteers increased as the 25(OH)D level decreased. To the best of our knowledge the present study is the first to evaluate the effect of vitamin D deficiency in healthy individuals. All previous studies on thyroid autoimmunity and vitamin D examined the prevalence of vitamin D deficiency in Hashimoto's thyroiditis. Recently, Tamer et al. investigated the prevalence of vitamin D deficiency in Hashimoto's thyroiditis patients and reported that there might be an association between Hashimoto's thyroiditis and vitamin D deficiency [16]. A study from Hungary reported a higher prevalence of vitamin D deficiency in patients with thyroid disease than in healthy controls. A community-based study from India evaluated 642 students and teachers aged 16–60 years and reported that the prevalence of anti-TPO was similar in the participants classified according to a serum 25(OH)D cut-off level of ≤25 nmol L^−1^ or >25 nmol L^−1^, but a weak inverse correlation between TPOAb titers and the serum 25(OH)D level was noted [17].

Currently, there is no consensus concerning the optimal serum 25(OH)D concentration during winter. The present study classified the participants according to 25(OH)D levels as follows: severe deficiency: <10 ng mL^−1^; moderate deficiency: 10–20 ng mL^−1^; normal: ≥20 ng mL^−1^. The parameters that could affect thyroid function or the vitamin D level, such as age, gender, BMI, insulin sensitivity, seasonal variation, and smoking status, were similar in all 3 groups; however, waist circumference did differ significantly between groups, but the literature contains no data linking waist circumference to the vitamin D level. In the present study the incidence of anti-TPO positivity was significantly higher in groups 1 and 2 than in group 3. Furthermore, the TSH level was lower in groups 1 and 2 than in group 3. There was only a significant weak inverse correlation observed between anti-TPO positivity and the 25(OH)D level, as in the Indian study mentioned earlier [17], and a positive correlation between the TSH level and the serum 25(OH)D level. In contrast to the Indian study [17], participants with a TSH level not in the euthyroid state were excluded from the present study.

The etiology of NTIS is multifactorial and recent studies show that inflammatory cytokines are responsible for the pathogenesis of the syndrome. The presence of low serum T3 and T4 levels is associated with morbidity and mortality in noncritically ill patients, such as those with heart disease and acute myocardial infarction [5]. The present study also determined the NTIS status in all participants, and only 1 individual in group 2 (moderate vitamin D deficiency) was NTIS positive. There was not a significant difference between the groups; however, our study population is limited for this investigation. Vitamin D receptor polymorphism is also important in evaluating the effect of vitamin D deficiency on thyroid autoimmunity, despite the existence of controversial reports of the absence of a role for functional vitamin D receptor polymorphism in the frequency of autoimmune thyroiditis [18–20].

The present findings show that more of the participants with vitamin D deficiency had anti-TPO positivity and lower TSH levels, as compared to those with a normal vitamin D level. These findings suggest that there might be a correlation between the severity of vitamin D deficiency and the occurrence of Hashimoto's thyroiditis. Additional large-scale studies are needed to more clearly determine if vitamin D deficiency plays a causal role in the pathogenesis of Hashimoto's thyroiditis and NTIS or if it is the result of disease.

## Figures and Tables

**Table 1:** Clinical characteristics of the participants according to group.

	Group 1 (severe vitamin D deficiency)	Group 2 (moderate vitamin D deficiency)	Group 3 (normal vitamin D level)	*P*
*n*	53	61	41	
Male/female	4/49	10/51	10/31	0.05
Current smokers (%)	40.0	27.0	22.2	>0.05
Age (years)	42.4 ± 2.0	37.9 ± 1.7	39.5 ± 1.9	>0.05
BMI (kg m^−2^)	26.1 ± 1.2	26.3 ± 1.0	24.9 ± 0.9	>0.05
WC (cm)	93.8 ± 2.9	91.0 ± 2.4	85.5 ± 2.5	0.04
FBG (mg dL^−1^)	82.7 ± 1.9	79.7 ± 1.6	83.4 ± 1.8	>0.05
HDL-C (mg dL^−1^)	51.1 ± 2.1	48.7 ± 1.9	47.1 ± 2.1	>0.05
LDL-C (mg dL^−1^)	112.7 ± 5.5	106.6 ± 4.9	112.1 ± 5.4	>0.05
TG (mg dL^−1^)	132.0 ± 9.0	114.1 ± 8.1	126.6 ± 8.8	>0.05
Fasting insulin (IU mL^−1^)	10.8 ± 1.2	9.4 ± 1.0	9.0 ± 1.1	>0.05
HOMA-IR (%)	2.1 ± 0.4	1.6 ± 0.4	1.5 ± 0.4	>0.05
TSH (mIU mL^−1^)	1.2 ± 0.15	1.5 ± 0.1	1.9 ± 0.1	0.008
FT4 (ng dL^−1^)	1.0 ± 0.0	1.1 ± 0.0	1.1 ± 0.0	>0.05
FT3 (ng dL^−1^)	2.8 ± 0.0	2.8 ± 0.0	2.7 ± 0.0	>0.05
Anti-TPO positivity (%)	62.7	71.7	24.2	0.000
Anti-Tg positivity (%)	21.6	21.7	25.6	>0.05

BMI: body mass index; WC: waist circumference; FBG: fasting blood glucose; HDL-C: high-density cholesterol; LDL-C: low-density cholesterol; TG: triglyceride.

**Table 2:** Characteristics of participants according to the vitamin cut-off level of 20 ng mL^−1^.

	<20 ng mL^−1^	≥20 ng mL^−1^	*P*
*n*	114	41	
Male/female	14/100	10/31	>0.05
Age (years)	39.7 ± 1.5	39.5 ± 1.9	>0.05
BMI (kg m^−2^)	26.2 ± 0.9	24.9 ± 0.9	>0.05
WC (cm)	92.1 ± 2.2	85.5 ± 2.5	0.02
FBG (mg dL^−1^)	80.9 ± 1.4	83.4 ± 1.8	>0.05
HDL-C (mg dL^−1^)	49.7 ± 1.6	47.1 ± 2.1	>0.05
LDL-C (mg dL^−1^)	109.1 ± 4.2	111.9 ± 5.3	>0.05
TG (mg dL^−1^)	121.7 ± 6.9	126.6 ± 8.8	>0.05
Fasting insulin (IU mL^−1^)	9.9 ± 0.9	9.0 ± 1.1	>0.05
HOMA-IR (%)	1.8 ± 0.4	1.5 ± 0.4	>0.05
TSH (mIU mL^−1^)	1.4 ± 0.1	1.9 ± 0.1	0.007
FT4 (ng dL^−1^)	1.1 ± 0.0	1.1 ± 0.0	>0.05
FT3 (ng dL^−1^)	2.8 ± 0.0	2.7 ± 0.0	>0.05

BMI: body mass index; WC: waist circumference; FBG: fasting blood glucose; HDL-C: high-density cholesterol; LDL-C: low-density cholesterol; TG: triglyceride.
